# LARIAT or AtriClip: Complications Profile and Comparison in Patients with Atrial Fibrillations Based on Manufacturer and User Facility Device Experience Database

**DOI:** 10.3390/medicina59122055

**Published:** 2023-11-21

**Authors:** Radosław Litwinowicz, Jakub Batko, Jakub Rusinek, Wojciech Olejek, Daniel Rams, Mariusz Kowalewski, Krzysztof Bartuś, Marian Burysz

**Affiliations:** 1 CAROL—Cardiothoracic Anatomy Research Operative Lab, Department of Cardiovascular Surgery and Transplantology, Institute of Cardiology, Jagiellonian University Medical College, 31-008 Krakow, Poland; 2 Department of Cardiac Surgery, Regional Specialist Hospital, 86-300 Grudziądz, Poland; 3 Thoracic Research Centre, Collegium Medicum Nicolaus Copernicus University, Innovative Medical Forum, 85-094 Bydgoszcz, Poland; 4 Department of Cardiac Surgery and Transplantology, National Medical Institute of the Ministry of Interior and Administration, Wołoska 137 Str., 02-507 Warsaw, Poland; 5 Cardio-Thoracic Surgery Department, Heart and Vascular Centre, Maastricht University Medical Centre, Cardiovascular Research Institute Maastricht (CARIM), 6229 HX Maastricht, The Netherlands; 6 Department of Cardiovascular Surgery and Transplantology, Institute of Cardiology, Jagiellonian University Medical College, 31-008 Krakow, Poland

**Keywords:** left atrial appendage, occlusion device, AtriClip, LARIAT, MAUDE, FDA

## Abstract

*Background and Objectives:* Left atrial appendage closure is an alternative treatment to reduce thromboembolism in patients with atrial fibrillation in whom oral anticoagulation (OAC) is contraindicated. The aim of this study was to evaluate the complications profiles of the LARIAT and AtriClip devices and perform a comparison between them based on the MAUDE (Manufacturer and User Facility Device Experience) database. *Materials and Methods:* The Manufacturer and User Facility Device Experience database was searched on 15 January 2023. For AtriClip, only reports regarding isolated procedures or procedures associated with minimally invasive ablation were included. Adverse effects and causes of death were defined based on the literature on the topic and the causes described in the reports. In total, 63 patients were included in the LARIAT group and 53 patients were included in the AtriClip group. *Results:* With the LARIAT device, the most common complication without device problems was pericardial effusion (*n* = 18, 52.9%), whereas this complication was not observed with AtriClip (*p* < 0.001). Postoperative bleeding was a second complication that occurred significantly more often in the LARIAT group—in 15 (44.1%) cases versus 1 (2.7%) case with AtriClip (*p* < 0.001). In addition, significant differences were found in the prevalence of stroke (LARIAT *n* = 0 vs. AtriClip *n* = 7, 18.9%, *p* = 0.012) and thrombus (LARIAT *n* = 2, 5.9% vs. *n* = 11, 29.7%, *p* = 0.013). *Conclusions:* Each type of left atrial appendage closure procedure is associated with device-specific requirements and complications that, if known, can be avoided.

## 1. Introduction

Left atrial appendage closure is an alternative treatment to reduce thromboembolism in patients with atrial fibrillation in whom oral anticoagulation (OAC) is contraindicated [1,2]. Occlusion of the left atrial appendage can be accomplished via two different approaches: endocardial or epicardial [3,4]. The endocardial approach to left atrial appendage closure procedures as a prevention and treatment strategy for atrial fibrillation and stroke has been demonstrated in numerous clinical trials and registries [5,6,7,8,9]. Epicardial procedures for left atrial appendage closure are reported in less extensive studies with limited data, which limits their application [10,11,12]. The 2020 European Society of Cardiology guidelines for the treatment of atrial fibrillation indicate that epicardial devices should be used, especially in atrial fibrillation patients who cannot tolerate antiplatelet agents [1]. Epicardial closure can be performed percutaneously with LARIAT devices or thoracoscopically with AtriClip devices.

The MAUDE database consists of reports of adverse events related to medical devices sent to the United States Food and Drug Administration (FDA). The reports can be exported. Each medical report contains a basic classification and a full description of the event.

Recent reports suggest a comparable risk profile and near-100% success of left atrial appendage closure with stable long-term outcomes [10,11,12,13,14]. However, there is no direct comparison between the two types of epicardial closure of the left atrial appendage for stroke prevention. In this study, we compare the adverse effects of left atrial appendage epicardial closure between the LARIAT and AtriClip devices based on the Manufacturer and User Facility Device Experience (MAUDE) database.

The purpose of this study was to provide a detailed description and comparison of the adverse effects of LARTIAT and AtriClip devices associated with left atrial appendage closure procedures during the period 2012–2022.

## 2. Materials and Methods

### 2.1. Search Strategy

A simple search for the word “atriclip” was performed for the AtriClip device, with the date of receipt of the report by the United States Food and Drug Administration set to the option “ALL YEARS”. The number of results was compared with the advanced search for “AtriClip” and was higher for the simple search. For the LARIAT device, the advanced search protocol developed by Jazayeri [15] was implemented and compared to the simple search for the words “LARIAT” and “SENTREHEART” with the date of the report received from the FDA set to the “ALL YEARS” option. The number of reports from the simple search was higher. The final search strategy was a simple search with the parameters described previously. The search was conducted on 15 January 2023.

### 2.2. Exclusion Criteria

The following exclusion criteria were applied in this study: duplication of reports, origin of reports from the literature or social media, insufficient information on adverse effects, and device-related technical complications with no impact on the patient (including preoperative damage to the device, damage to the cover, etc.). For AtriClip, only reports regarding isolated procedures or procedures associated with minimally invasive ablation were included. In reports where insufficient information was reported on device-related problems, this was treated as not being a device-related problem.

### 2.3. Evaluation of Reports

Duplicate reports were first removed through an automatic function in Microsoft^®^ Excel v.16.67 software (Microsoft Corporation, Redmond, WA, USA). Subsequently, all reports were manually analyzed by two independent researchers to evaluate report origin, adverse effects, and cause of death. Additional duplicates found during reading were manually removed.

### 2.4. Adverse Effects and Cause of Death

Adverse effects and causes of death were defined based on the literature on the topic and the causes described in the reports. If the cause of death or adverse effect reported by both investigators was different in a report, the final cause of death or adverse effect was determined by the supervisor. In the case of perforation, the location of the adverse effect and further treatment were analyzed, if described. For a pericardial effusion, the additional cause and further treatment were analyzed.

### 2.5. Number of Patientsanalyzed

For the LARIAT device, 63 patients were considered for statistical analysis (180 exported, 82 duplicates, 35 met exclusion criteria). For the AtriClip device, 53 patients were included for statistical analysis (280 exported, 39 duplicates, 202 met exclusion criteria (186 reports of ineffective device malfunction, 16 reports from social media and the literature)).

### 2.6. Statistical Analysis

Categorical variables were presented as numbers and percentages. Chi-square test for independence or Fisher’s exact test were performed to detect differences in categorical variables. Differences between adverse events and causes of death were evaluated. Statistical analyses were performed using IBM SPSS Statistics 29.0 (Predictive Solutions, Armonk, NY, USA). A *p* value of less than 0.05 was considered statistically significant.

## 3. Results

### 3.1. Prevalence of Device Problems and Adverse Effects in Patients

Device problems occurred in 29 (46.0%) reports for LARIAT and in 16 (30.2%) reports for AtriClip. Overall, 5 (11.1%) patients with device malfunction died, 13.8% for LARIAT (4/29) and 6.3% for AtriClip (1/16) (*p* = 0.440). The causes of death were perforation of the left atrial appendage (two cases), hemorrhage of unknown cause (two cases), and one case with unknown cause of death.

### 3.2. Adverse Events in Patients with Device Problems

When comparing the LARIAT and AtriClip devices and adverse events in patients with device problems, there was only a statistically significant difference in the prevalence of the adverse event—pericardial effusion, which occurred in 11 (37.9%) patients with device problems with LARIAT and 0.0% of patients with device problems with AtriClip (*p* = 0.004). There was no statistically significant difference between other adverse effects. Detailed information on complications in patients with device problems is presented in Table 1.

### 3.3. Adverse Side Effects in Patients without Device Problems

With the LARIAT device, the most common complication without device problems was pericardial effusion (n = 12, 52.9%), whereas this complication was not observed with AtriClip (*p* < 0.001). Postoperative bleeding was a second complication that occurred significantly more often in the LARIAT group—in 15 (44.1%) cases versus 1 (2.7%) case with AtriClip (*p* < 0.001). In addition, significant differences were observed in the prevalence of stroke (LARIAT 0.0% vs. AtriClip n = 7, 18.9%, *p* = 0.012) and thrombus (LARIAT n = 2, 5.9% vs. AtriClip n = 11, 29.7%, *p* = 0.013). Detailed information can be found in Table 2.

### 3.4. Perforation

**Figure 1:**
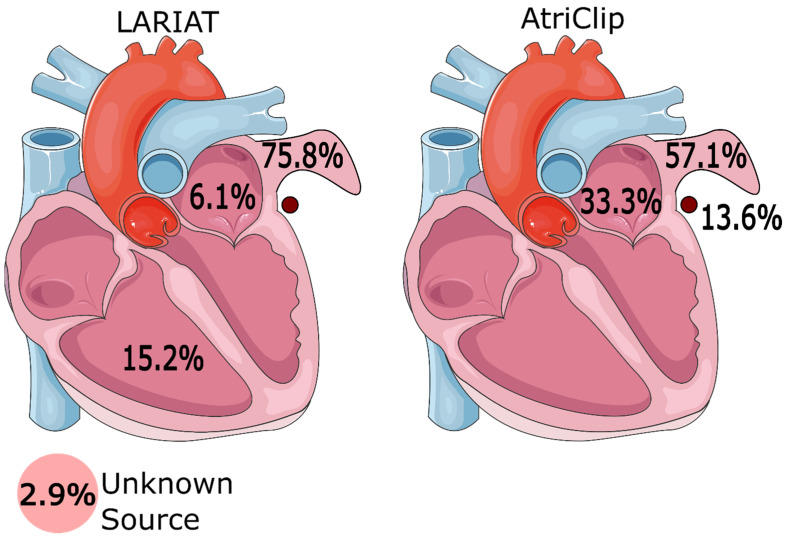
Illustration of perforation location. Additionally, the prevalence of left circumflex artery occlusion was considered.

Perforation was reported in 52.4% (33/63) of cases for LARIAT versus 39.6% (21/53) for AtriClip. The most common perforation localizations for the LARIAT device were left atrial appendage (n = 25, 75.8%), right ventricle (n = 5, 15.2%), and left atrium (n = 2, 6.1%). The most common perforation localizations for the AtriClip device were left atrial appendage (n = 12, 57.1%), left atrium (n = 7, 33.3%), and left circumflex artery (including occlusion) (n = 3, 13.6%). The most common treatments for perforation with the LARIAT device were cardiac surgery (n = 20, 60.6%), pericardiocentesis (n = 5, 15.2%), and blood transfusion (n = 5, 15.2%). The most common treatments for perforation with the AtriClip device were repair without advancing the procedure (n = 8, 38.1%), sternotomy (n = 7, 33.3%), and use of a second AtriClip (n = 3, 14.3%). Detailed perforation information is shown in Figure 1.

### 3.5. Pericardial Effusion

Pericardial effusion was reported exclusively in patients who underwent left atrial appendage closure with the LARIAT device. Tamponade was observed in two patients. Cardiopulmonary resuscitation was required in an additional two cases. No cases of pericarditis were observed. The most common procedures for the treatment of pericardial effusion were open heart surgery (n = 24, 82.8%) and pericardiocentesis (n = 16, 55.2%).

### 3.6. Thrombus

All cases of thrombus formation after the LARIAT procedure were observed in pericardial suction after cardiac perforation and were associated with postoperative bleeding. Thirteen thromboses were observed in the AtriClip group, including five with unspecified location. The most common thrombus localizations were the left atrial appendage (five cases) and the left atrium (two cases). In one case, thrombus was reported in the lower extremities. Four cases underwent therapeutic intervention to treat the thrombus, including cardiac surgery (two cases), classic surgery (one case), and thrombus aspiration (one case).

### 3.7. Cause of Death

Death was reported in 11 (17.5%) cases with patient problems in the LARIAT group and in 8 (15.1%) in the AtriClip group. The most common cause of death in the LARIAT group was left atrial appendage perforation (in six cases (54.5%)), and in the AtriClip group, stroke was the most common cause of death (in two cases (25%)). In four cases (21.1%), the patient’s cause of death was unknown. Detailed information on the cause of death of patients can be found in Table 3.

## 4. Discussion

### 4.1. MAUDE Database Results Compared to Historical Findings

The MAUDE database results related to the complication of left atrial appendage occlusion should be analyzed with caution, particularly for cases that do not meet United States Food and Drug Administration requirements for reporting all adverse effects. However, the complications reported in it serve as a source of complications that have not been observed in randomized controlled trials on this topic [10,11,12,13,14]. Interestingly, our study found a lower rate of complications related to device malfunction than complications unrelated to the device. Pericardial effusion, which was observed exclusively with the LARIAT procedure, may be related to the minimally invasive nature of the procedure, in which the AtriClip is usually implanted during another procedure. One of the most common complications of epicardial occlusion of the left atrial appendage closure is tissue perforation. The distribution of perforation sites differs between the LARIAT and AtriClip procedures, mainly because of the technical aspects of the procedure—the LARIAT procedure requires percutaneous localization of loop navigation instruments, resulting in potential additional tissue damage to structures adjacent to the AtriClip route, including the arterial access point, right heart structures, and, eventually, the left atrial appendages and left atrium. On the other hand, the AtriClip more often results in damage to structures adjacent to the left atrial appendage. This may be caused by mispositioning of the clip after implantation, leading to rupture or occlusion of the left atrium or nearby left coronary artery. Several studies were performed to evaluate the efficacy and safety of the LARIAT procedure [11,13,14]. No device-related complications were reported, with access-related complications ranging from 3 to 5%. Mortality was 0–5%, and stroke was observed in 0–3.7% of patients. The most frequently observed complication was pericardial effusion, ranging from 1% to 15%. Major bleeding was observed in only one patient in only one study. Two large prospective studies of the AtriClip procedure for left atrial appendage closure have been published [10,12]. In the first study, in which the AtriClip device was used in conjunction with open heart surgery, serious adverse events occurred in 48.6% of patients, whereas in the second study, a serious adverse event was observed in 0.27% of patients. Such a difference may be attributed to the different procedures performed during open heart surgery, which may be associated with such a variation in complication rates.

### 4.2. Potential Prevention of the Surgical Complications

In our study, we found that one of the most commonly reported complications of left atrial appendage closure was perforation. Proper preoperative visualization combined with the surgeon’s clinical experience plays a crucial role in preventing such complications [16,17]. The anatomy of cardiac structures is still being explored, with recent significant clinical findings having a positive impact on the safety of cardiac surgery and procedures tailored to the patient. One of the rare but fatal clinical complications of left atrial appendage occlusion, which is closely related to its anatomic topography, is occlusion of the left circumflex artery [18,19]. Its frequency is difficult to determine, but we found at least three described cases in the MAUDE database, one of which was fatal to the patient. Left coronary artery manipulations should be performed with great caution, especially when the left atrial appendage and left circumflex artery are in an unfavorable relationship. Our results support previous suggestions that to avoid complications from left circumflex artery occlusion during left atrial appendage occlusion, the LARIAT system should be used [16].

### 4.3. Left Atrial Appendage Closure Procedure Complications

Iatrogenic cardiac perforation is a rare but potentially fatal complication associated with invasive cardiac procedures [20]. The most common surgical approach for its treatment is sternotomy, as it provides easy and complete access to the potential source of bleeding when it cannot be located preoperatively. However, a proof-of-concept study recently published by Langenaeken and coworkers shows that the use of video-assisted thoracoscopic surgery offers potential advantages in such cases [20]. This interesting topic should be further investigated in a larger patient population in the future to demonstrate its clinical safety and efficacy. Postoperative thromboembolism is a serious complication that can occur in patients undergoing surgery for a variety of reasons [21,22]. Cardiovascular surgery is associated with an increased risk of thrombus formation. Among cardiac surgical procedures, coronary artery bypass grafting was associated with the highest risk of thrombus formation of 5.2%, whereas for other cardiac surgical procedures, the risk of thrombus formation was 4.3%. Treatment with anticoagulants reduced this risk by about half to one percent [21]. These complications were last observed within one month after surgery. Pericardial effusion is estimated to be found in approximately 1.5% of patients after cardiac surgery, with nearly half of them having concomitant cardiac tamponade. In their retrospective study, Ashikhmina and coworkers found that the most common treatment for pericardial effusion was echocardiographically guided pericardiocentesis (in more than 50% of cases) and surgical drainage (in 23% of cases) [23]. Conservative treatment was the therapeutic approach in one fifth of patients. In addition, some risk factors for pericardial effusion were identified, including obesity, hypertension, renal failure, and prolonged cardiopulmonary bypass. Interestingly, previous cardiac surgery was associated with a lower risk of pericardial effusion.

### 4.4. Treatment of Atrial Fibrillation

Atrial fibrillation is the most commonly diagnosed cardiac arrhythmia, affecting an estimated 1–2% of the adult population. Atrial fibrillation leading to ischemic complications is independently associated with an increased risk of death [24]. There are many methods for atrial fibrillation treatment, such as endocardial or epicardial ablation or left atrial appendage closure for thromboprophylaxis [1].

### 4.5. The Clinical Anatomy of the Left Atrial Appendage

The left atrial appendage is a small, muscular structure located inferior to the left superior pulmonary vein and adjacent to major anatomic structures, including the left circumflex artery, left coronary artery, aorta, pulmonary truncus, and mitral valve [25,26]. Clinically, it can be divided into the neck, which contains the landing zone for occlusions and occluding devices, and the body, which contains the thick network of pectinate muscles [16]. To date, several classifications of the left atrial appendage have been developed, including the most recent simplified classification [26]. However, these classifications focus on the relationship between the shape of the body and the risk of thrombus formation, and do not discuss the effects of anatomic shape on occlusion implantation and landing zone location. It has already been established that the morphometric characteristics of the left atrial appendage differ between the healthy population and patients with atrial fibrillation [27]. However, the long-term anatomic effects of atrial fibrillation on the anatomy of the left atrial appendage remain to be determined.

### 4.6. Roles of the Left Atrial Appendage

The left atrial appendage plays a crucial role as the main thrombus formation source in patients with atrial fibrillation. Additionally, recent studies proved the crucial impact of the left atrial appendage on the regulation of systemic coagulation factors [28,29]. New important systemic functions of the left atrial appendage are now being described. The four major areas of influence have been defined by Alkhouli and coworkers: hormonal regulation, hemodynamics, atrial arrhythmia, and stem cell reservoir [30]. Neurohormonal regulation is related to changes in ANP and BNP secretion (which may be affected by epicardial occlusion of the left atrial appendage closure, as demonstrated in the HOMEOSTASIS study) [31] and, in addition, to the long-term regulation of aldosterone, epinephrine, norepinephrine, renin, and vasopressin, the levels of which were persistently and significantly reduced in patients undergoing left atrial appendage closure with the LARIAT device in the HOMEOSTASIS II study [32,33,34]. The hemodynamic effects of the left atrial appendage have been demonstrated in several studies, including the LAFIT study, which demonstrated a potential beneficial effect on the left atrium after left atrial appendage closure, but its long-term effects should be further investigated [35]. The arrhythmogenic role of the left atrial appendage was first described by Di Biase and colleagues, who found that 27% of premature atrial contractions in patients requiring redo catheter ablation of AF originated from the left atrial appendage [36]. In this context, the BELIEF study (Left Atrial Appendage Isolation in Patients with Longstanding Persistent Atrial Fibrillation Undergoing Catheter Ablation) was performed [37]. This demonstrated that extensive ablation of the left atrial appendage in these cases decreases the recurrence rate of AF by almost two times. Last but not least, the left atrial appendage is a stem cell reservoir that can be used as a valuable source for cardiac stem cell therapy. Initial promising results include a reduction in oxidative stress and increased reparative capacity of cardiomyocytes [38,39]. Despite these not yet fully understood but clinically important functions of the left atrial appendage, new roles associated with the anatomical localization of the left atrial appendage continue to be discussed.

### 4.7. The Left Atrial Appendage Occlusion Procedures

Left atrial appendage closure and occlusion procedures can be divided into two main types: epicardial or endocardial [1,4]. Epicardial procedures are based on external occlusion of the left atrial appendage neck closure with a loop (LARIAT) or clip (AtriClip) [10,11,12,13,14]. The LARIAT system consists of the following. (A) A 0.025-inch endocardial guidewire with magnetic tip, which is placed in the left atrium by inserting a catheter into the femoral vein. (B) A 0.035-inch magnetic-tipped guidewire at the epicardium, which is inserted into the atrial appendages on the outside of the left atrium by puncturing the pericardium. Each wire has an opposite-polarity magnet that allows end-to-end alignment. (C) A 15 mm compliant occlusion balloon catheter to identify the left atrial appendage and allow very precise and effective occlusion of the left atrial appendage. (D) The LARIAT suture delivery device, which is inserted through a prior puncture of the pericardium near the left atrial appendage. The LARIAT suture device is used to close the lumen of the left atrial appendage from the outside to eliminate the source of the thrombus. The patient is placed in the supine position and intubated under general anesthesia, and the TEE probe is inserted. The LARIAT procedure is performed with three main components: a compliant occlusion balloon, two magnet-tipped guidewires and a 12-Fr suture device. After percutaneous pericardial access, a transseptal puncture is performed. The first endocardial magnet-equipped guidewire is placed near the apex of the left atrial appendage. Through a percutaneous femoral approach, the second endocardial magnet-equipped guidewire is placed at the tip of the left atrial appendage to establish a stable connection between the wires. The LARIAT snare is then advanced over the epicardial guidewire to occlude the left atrial appendage. After TEE and fluoroscopic confirmation of left atrial appendage closure, a pre-tied suture is placed and tightened to ligate the left atrial appendage. AtriClip (AtriClip PRO Device, AtriCure, Dayton, OH, USA) is designed for complete thoracoscopic closure of the left atrial appendage [12]. The AtriClip device consists of an automatically closing clip placed in a deployment loop on a disposable holder whose head is movable 60 degrees laterally and up/down. The novel features of the system compared to previous systems, such as length, maneuverability, and deployment system, allow for a fully thoracoscopic application. The patient is positioned supine and intubated under general anesthesia with a double-lumen intratracheal tube. A transesophageal echocardiography probe is inserted. Ventilation of the right lung is initiated. Then, three thoracoscopic ports are placed, one through the fourth intercostal space in the anterior axillary line (for the endoscopic camera) and two through the third and sixth intercostal spaces in the middle axillary line into the left pleura (working ports). The working space is created with CO_2_ insufflation. Pericardiectomy is performed parallel to the phrenic nerve to visualize the left atrial appendage. Holding sutures are placed at the inferior border of the pericardium to gain better access to the left atrial appendage. The diameter of the base of the left atrial appendage is measured using a special selection guide. The AtriClip device is inserted through the incision in the sixth intercostal space, which is enlarged to 2–3 cm. Under the control of TEE, the AtriClip is attached to the base of the left atrial appendage, with special care taken not to leave a stump. The possibility of repeated opening of the clip before final deployment allows correction of its position if intraoperative echocardiography shows incomplete occlusion of the left atrial appendage or residual left atrial appendage. Complete left atrial appendage exclusion is then confirmed through visualization at TEE.

Epicardial procedures are usually performed as part of other cardiac surgical procedures because they require epicardial access to the left atrial appendage, which in other cases can be achieved during thoracoscopy. Endocardial closure of the left atrial appendage is a percutaneous procedure in which the plug is inserted into the neck of the left atrial appendage. This procedure requires puncture of the atrial septum and may be associated with additional complications as the plug moves after implantation [40]. Occlusion of the left atrial appendage reduces the risk of thromboembolism by eliminating the most common site of thrombus formation in the human heart. In addition, changes in fibrinolytic activity and coagulation factors were observed in patients treated with epicardial occlusion of the left atrial appendage. Thus, the impact of this procedure is multidimensional and provides major health benefits for patients with atrial fibrillation [28,29].

### 4.8. Limitations

Our study is based on the mandatory reports sent to the MAUDE database, so the actual incidence of the analyzed complications cannot be determined. In addition, because of the limited search time, only a 10-year period can be exported from the MAUDE database. The patients’ demographic characteristics are not collected in this repository, so the impact of comorbidities on postoperative complications cannot be assessed.

## 5. Conclusions

Left atrial appendage closure is a safe and efficient method to prevent ischemic complications in patients with AF. Each type of left atrial appendage closure procedure is associated with device-specific requirements and complications that, if known, can be avoided.

## Figures and Tables

**Table 1:** Complications coexisting with device problems.

Parameter	LARIAT *n* (%) (*n* = 29)	AtriClip *n* (%) (*n* = 16)	*p*-Value
Arrythmia	0 (0.0%)	1 (6.3%)	0.356
Cardiac arrest	0 (0.0%)	1 (6.3%)	0.356
Postoperative bleeding	14 (48.3%)	4 (25.0%)	0.127
Hypotension	3 (10.3%)	1 (6.3%)	1.000
Inflammation	1 (3.4%)	0 (0.0%)	1.000
Thrombus	2 (6.9%)	2 (12.5%)	0.608
Perforation	16 (55.2%)	7 (43.8%)	0.463
Pericardial effusion	11 (37.9%)	0 (0.0%)	0.004

**Table 2:** Complications without device problems.

Parameter	LARIAT *n* (%) (*n* = 34)	Atriclip *n* (%) (*n* = 37)	*p*-Value
Arrythmia	0 (0.0%)	1 (2.7%)	1.000
Cardiac tamponade	2 (5.9%)	2 (5.4%)	1.000
Cardiomyopathy	0 (0.0%)	1 (2.7%)	1.000
Endocarditis	0 (0.0%)	1 (2.7%)	1.000
Heart block	0 (0.0%)	1 (2.7%)	1.000
Heart failure	0 (0.0%)	1 (2.7%)	1.000
Hematoma	0 (0.0%)	1 (2.7%)	1.000
Postoperative bleeding	15 (44.1%)	1 (2.7%)	<0.001
Hypotension	7 (20.6%)	2 (5.4%)	0.077
Perforation	15 (44.1%)	14 (37.8%)	0.590
Pericardial effusion	18 (52.9%)	0 (0.0%)	<0.001
Pleural effusion	1 (2.9%)	0 (0.0%)	0.479
Stroke	0 (0.0%)	7 (18.9%)	0.012
Thrombus	2 (5.9%)	11 (29.7%)	0.013
Vascular dissection	0 (0.0%)	3 (8.1%)	1.000

**Table 3:** Patients’ cause of death. LAA—left atrial appendage, PEA—pulseless electrical activity, Cx—left circumflex artery.

Cause of Death	LARIAT *n* (%), *n* = 11	AtriClip *n* (%), *n* = 8	*p*
unknown	3 (27.3%)	1 (12.5%)	0.435
LAA perforation	6 (54.5%)	1 (12.5%)	0.060
PEA	1 (9.1%)	0 (0%)	1.000
hemorrhage	1 (9.1%)	1 (12.5%)	0.811
stroke	0 (0%)	2 (25%)	1.000
Cx occlusion	0 (0%)	1 (12.5%)	1.000
sepsis	0 (0%)	1 (12.5%)	1.000
heart failure	0 (0%)	1 (12.5%)	1.000

## Data Availability

The data from the study are available upon reasonable request from the corresponding author.
